# Follicle Size Regulates Cytoplasmic Maturation, Lipid Content, and Embryo Cryosurvival in Bovine Oocytes Matured In‐Vitro

**DOI:** 10.1002/mrd.70142

**Published:** 2026-07-29

**Authors:** Briza Castro, Paula Rodriguez‐Villamil, M. Sofia Ortega

**Affiliations:** ^1^ Department of Animal and Dairy Sciences University of Wisconsin‐Madison Madison Wisconsin; ^2^ ABS Global DeForest Wisconsin USA

**Keywords:** embryo cryosurvival, follicle size, lipid metabolism, Oocyte competence

## Abstract

In‐vitro embryo production (IVP) in cattle is limited by the suboptimal competence of oocytes matured outside the follicular environment. While maturation is often achieved, incomplete cytoplasmic maturation may impair embryo development and survival. This study evaluated the influence of follicle size on oocyte cytoplasmic maturation, lipid metabolism, cumulus cell function, and embryo cryosurvival. Oocytes were collected from small (< 6 mm), medium (6–9 mm), and large (10–20 mm) follicles and evaluated at 0, 12, and 24 h of in‐vitro maturation (IVM). Organelle distribution, lipid and mitochondrial content and distribution, cortical granule distribution, and transzonal projections (TZPs) were evaluated. Gene expression was assessed in surrounding cumulus cells, and embryo development and cryotolerance were evaluated following fertilization and culture. While organelle patterns did not differ among follicle sizes for mitochondria, lipids, or cortical granules, oocytes from large follicles had lower lipid accumulation by 24 h IVM, and fewer TZPs throughout maturation. Gene expression in cumulus cells from large follicles also had a greater abundance of transcripts involved in steroidogenesis, growth factor signaling, and glucose metabolism midway through maturation. Although cleavage and blastocyst development rates were similar across groups, embryos from large follicle oocytes had higher post‐thaw survival and lower lipid content than those from small follicles. These findings demonstrate that follicle size affects key metabolic and structural aspects of oocyte maturation, ultimately influencing embryo quality. Selecting oocytes from larger follicles may improve embryo cryosurvival by promoting more complete cytoplasmic maturation and more efficient lipid regulation during IVM.

## Graphical abstract

Follicle size modulates bovine oocyte cytoplasmic maturation during IVM. Oocytes from larger follicles exhibit reduced lipid accumulation, fewer transzonal projections, distinct cumulus cell metabolic signaling, and greater embryo cryosurvival, indicating improved cytoplasmic maturation and lipid regulation despite similar developmental rates.

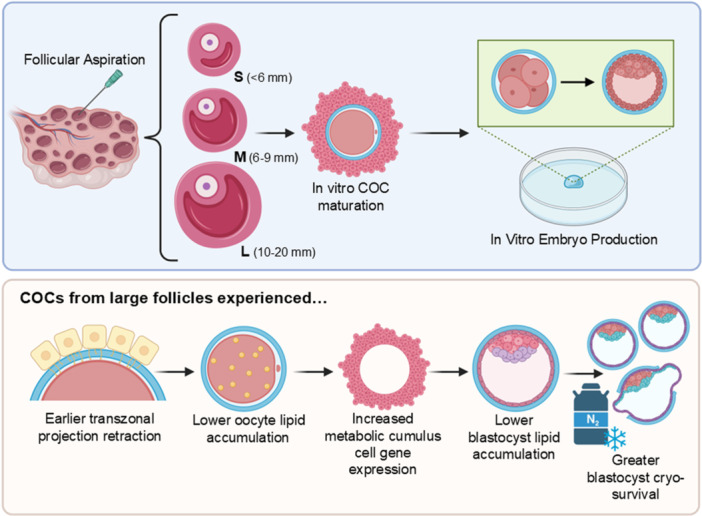

## 1 Introduction

Despite advances in assisted reproductive technologies (ARTs), the efficiency of in‐vitro embryo production (IVP) in cattle remains suboptimal. While up to 80% of oocytes undergo successful nuclear maturation, only 20%–30% develop into transferable embryos (Ferré et al. 2020). By contrast, embryos derived in‐vivo consistently exhibit superior quality and developmental outcomes (Viana 2023). One key factor limiting IVP efficiency is incomplete or suboptimal oocyte maturation (Pytel et al. 2024), comprising nuclear and cytoplasmic changes, both of which are critical for fertilization and embryo development.

Although nuclear maturation has long served as a convenient proxy for oocyte competence, cytoplasmic maturation is equally essential. During follicular development and before ovulation, the oocyte undergoes extensive cytoplasmic remodeling, including organelle replication and redistribution, accumulation of metabolic substrates, and active communication with surrounding cumulus cells via transzonal projections (TZPs). These projections facilitate the transport of mRNA, proteins, lipids, and metabolites necessary for oocyte maturation. Organelle dynamics, such as mitochondrial and cortical granule redistribution, reflect the developmental potential of the oocyte. In competent oocytes, mitochondria are evenly distributed throughout the cytoplasm, facilitating ATP production for meiotic progression and early embryogenesis (Machatkova et al. 2012; Reader et al. 2017; Stojkovic et al. 2001). Similarly, cortical granules transition from dispersed to peripheral distribution to support the cortical reaction and prevent polyspermy after fertilization (Hosoe and Shioya 1997).

Bovine oocytes reach their full diameter of approximately 120 µm when follicles are 3–4 mm in diameter (Alam et al. 2018); however, oocyte size alone does not equate to developmental competence. Oocytes from follicles > 6 mm exhibit greater developmental competence compared to those from smaller follicles (Brevini Gandolfi and Gandolfi 2001; Dadarwal et al. 2015). In‐vivo, oocyte maturation occurs in dominant follicles of approximately > 15 mm, culminating in ovulation post‐LH surge (Driancourt 1991). In contrast, research utilizing slaughterhouse‐derived ovaries for IVP often yields oocytes from small to medium follicles (1–6 mm), which produce fewer blastocysts than in‐vivo matured counterparts (Lonergan et al. 2006). Follicle size has long been recognized as a determinant of bovine oocyte competence, with oocytes recovered from follicles ≥ 6 mm consistently exhibiting improved developmental outcomes compared to those from smaller follicles (Carolan et al. 1996; Humblot et al. 2005; Lequarre et al. 2005; Lonergan et al. 1994; Machatkova et al. 2004; Sarwar et al. 2020).

Despite this extensive body of work, most studies have focused on cleavage and blastocyst yield as endpoints, with limited attention to the timing and coordination of cytoplasmic maturation events during in‐vitro maturation (IVM) or their relationship to embryo cryotolerance. As a result, mechanistic links between follicle size, oocyte cytoplasmic quality, and post‐embryonic resilience remain poorly defined. The present study builds on this foundational work by resolving how follicle size influences mitochondrial redistribution, lipid dynamics, and transzonal projection remodeling across defined maturation intervals, and by linking these processes to post‐thaw blastocyst survival rather than developmental rates alone.

While oocyte source heterogeneity and standardized culture conditions are inherent in most IVP systems, the extent to which follicle size alone programs cytoplasmic maturation under in‐vitro conditions remains unclear. Given that cytoplasmic changes are essential for embryonic development, understanding how follicular origin shapes oocyte metabolic readiness is critical for optimizing IVP protocols. Therefore, the objective of this study was to determine how follicle size influences cytoplasmic maturation, organelle dynamics, and subsequent embryo development in bovine oocytes matured in‐vitro. It was hypothesized that follicle size impacts cytoplasmic maturation, including organelle distribution, lipid content, and embryo development, even under standardized IVM conditions.

## 2 Materials and Methods

### 2.1 Ethics Statement and Tissue Information

Ovaries were obtained as a byproduct of commercial slaughter, and no animals were euthanized for this study. Therefore, institutional animal care and use committee approval was not required. Ovaries were collected from cattle at commercial slaughterhouses (American Foods Group, Green Bay, WI; JBS, Green Bay, WI). Specific breed composition and genetic information were not available from the processing facilities. Ovaries were obtained as pools from each collection with follicles across all three size categories represented within each collection. Ovaries with gross pathological abnormalities were excluded; however, the presence of corpora lutea was not used as an exclusion criterion, as this reflects normal cyclicity in unsynchronized cattle. As a result, follicle stage (e.g., growing vs. dominant vs. atretic) could not be formally classified, which represents an inherent limitation of the slaughterhouse IVP model.

### 2.2 Cumulus Oocyte Complex Collection

Ovaries were transported from a slaughterhouse at room temperature. Upon arrival, ovaries were washed with pre‐warmed 0.9% NaCl containing 1% Penicillin‐Streptomycin (Sigma‐Aldrich, St Louis, MO, USA) to remove excess blood. Cumulus oocyte complexes were aspirated from small (< 6 mm), medium (6–9 mm), and large (10–20 mm) follicles. Follicle size categories (< 6 mm, 6–9 mm, and 10–20 mm) were selected based on established classifications in bovine IVP studies, distinguishing growing, selection‐stage, and preovulatory‐sized follicles (Hendriksen et al. 2000; Lonergan et al. 1994; Machatkova et al. 2004).

Aspiration was done using an 18‐gauge needle and vacuum pressure of approximately 100 mmHg into separate 50 mL conical tubes containing oocyte collection medium composed of Medium‐199 with Earle's Balanced salts and l‐glutamine (Sigma Aldrich), 10.0 mM HEPES (Sigma Aldrich), and 4.16 mM sodium bicarbonate (Sigma Aldrich), supplemented with 0.5% glutaMAX (Thermo Fischer Scientific, Waltham, MA, USA), 2% adult bovine serum (Fisher Scientific), 2% Penicillin‐Streptomycin, and 2 U/mL heparin (Sigma Aldrich). Only COCs from small, medium, and large follicles with at least three layers of compact cumulus cells and uniform cytoplasm were selected.

### 2.3 IVM of Oocytes

Oocyte cytoplasmic maturation was evaluated at 0, 12, and 24 h of IVM. Briefly, oocyte maturation medium was composed of Tissue Culture Medium‐199 with Earle's salts (Gibco, Grand Island, NY, USA) supplemented with 10% fetal bovine serum (Sigma Aldrich), 2 µg/mL estradiol‐17β (Sigma Aldrich), 25 µg/mL Porcine purified follicle‐stimulating hormone (FSH; Folltropin; Vetoquinol, Fort Worth, TX, USA), 22 µg/mL sodium pyruvate (Sigma Aldrich), 50 µg/mL gentamicin (Gibco), and 1 mM glutaMAX (Tríbulo et al. 2019). Cumulus oocyte complexes were matured in 5‐well plates (WTA, São Paulo, Brazil) in 500 µL pre‐equilibrated maturation media overlaid with mineral oil (FUJIFILM Irvine Scientific, Santa Ana, CA, USA) and matured at 38.5°C, 5% CO_2_, and humidified air. Cumulus oocyte complexes from small, medium, and large follicles were matured in groups of 25‐30 COCs/well, with an individual well plated per timepoint and size. Wells were collected at 0, 12, or 24 h of maturation. At the time of collection, cumulus cells were mechanically removed by pipetting with a stripper tip and stored at −80°C for subsequent RNA isolation, and oocytes were used for fluorescence microscopy.

### 2.4 Oocyte Mitochondria, Lipids, Cortical Granules, and TZPs Characterization

**Figure 1:**
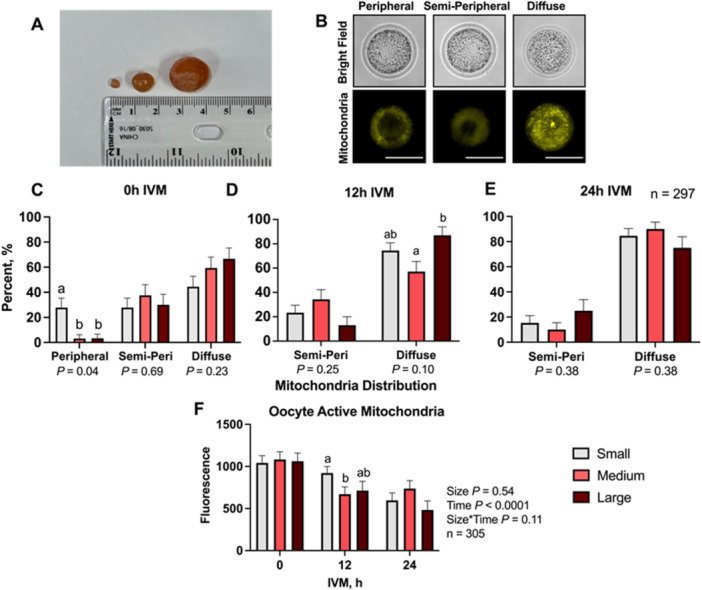
Active mitochondria distribution in oocytes derived from small, medium, and large follicles during IVM. (A) Representative images of bovine follicles categorized as small (< 6 mm), medium (6–9 mm), and large (10–20 mm). (B) Mitochondrial distribution patterns are classified as peripheral, semi‐peripheral, or diffuse. (C–E) Proportion of oocytes displaying each mitochondrial distribution pattern at 0 (C), 12 (D), or 24 (E) h IVM (*n* = 297 oocytes; ~33 oocytes per follicle size/timepoint). Oocytes from small follicles exhibited greater peripheral mitochondrial distribution at 0 h compared to medium and large follicles (*p* = 0.03). At 12 h, oocytes from large follicles displayed more diffuse mitochondrial distribution than those from medium follicles (*p* = 0.03). No differences in distribution patterns were observed among follicle sizes at 24 h (*p* > 0.05). (F) Quantification of active mitochondria at 0, 12, and 24 h IVM. At 12 h, oocytes from medium follicles had reduced mitochondrial membrane potential compared to those from small follicles (*p* = 0.02); no other differences were observed (*n* = 305 oocytes; ~33 oocytes per follicle size/timepoint). Scale bar = 100 µm.

**Figure 2:**
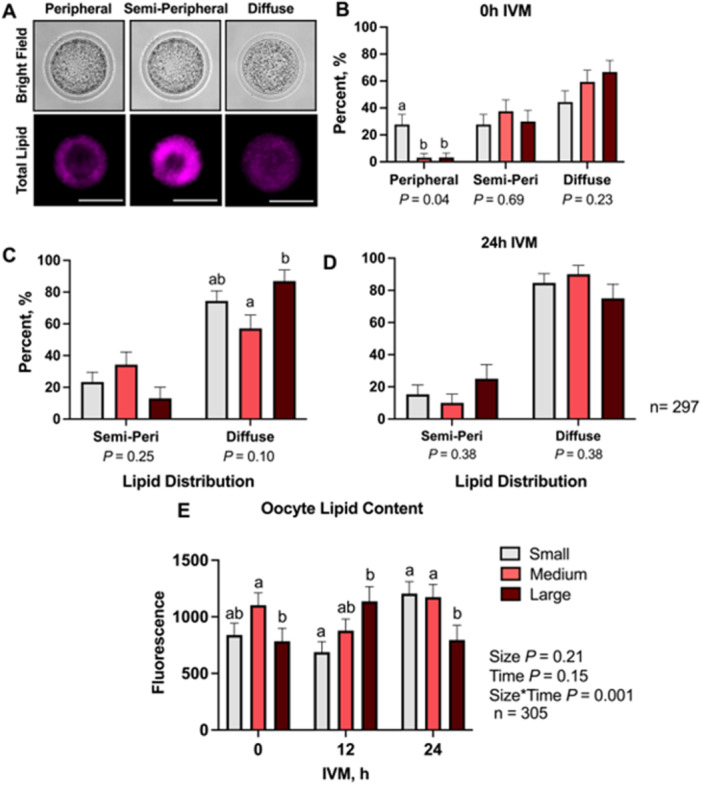
Lipid droplet distribution and content in oocytes derived from small, medium, and large follicles during IVM. (A) Representative images of lipid droplet distribution patterns classified as peripheral, semi‐peripheral, or diffuse. (B–D) Percentage of oocytes exhibiting each lipid distribution pattern at 0 (B), 12 (C), and 24 (D) h IVM (*n* = 297 oocytes; ~33 oocytes per follicle size/timepoint). Oocytes from small follicles had a greater percentage of peripheral lipid distribution at 0 h compared to medium and large follicles (*p* = 0.03). At 12 h, oocytes from large follicles had more diffuse lipid distribution than those from medium follicles (*p* = 0.03). No differences in lipid distribution were observed among follicle sizes at 24 h (*p* > 0.05). (E) Quantification of lipid droplet content in oocytes at 0, 12, and 24 h IVM (*n* = 305 oocytes; ~33 oocytes per follicle size/timepoint). At 0 h, oocytes from large follicles had lower lipid content compared to those from medium follicles (*p* = 0.02), but not from small follicles (*p* = 0.73). At 12 h, lipid content was higher in oocytes from large follicles compared to those from small (*p* = 0.07). By 24 h, oocytes from large follicles exhibited the lowest lipid content compared to medium (*p* = 0.04), and small (*p* = 0.008) follicle groups. Scale bar = 100 µm.

**Figure 3:**
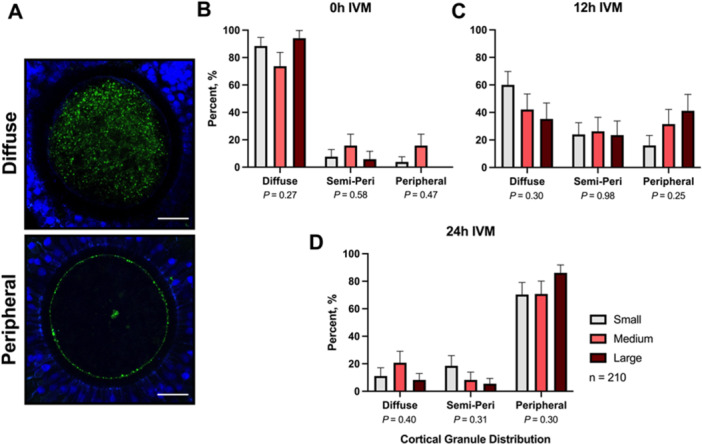
Cortical granule distribution in oocytes from small, medium, and large follicles during IVM. (A) Representative images of cortical granule distribution patterns classified as diffuse or peripheral. (B–D) Percentages of oocytes displaying each cortical granule pattern at 0 (B), 12 (C), and 24 (D) h IVM (*n* = 210 oocytes; ~23 oocytes per follicle size/timepoint). No significant differences in cortical granule distribution were observed among follicle size groups at the onset of maturation (*p* > 0.05). Although granules were gradually redistributed to the cortex by 12 h, differences among follicle sizes were not detected at any time point (*p* > 0.05). Scale bar = 20 µm.

To localize mitochondria, following the respective maturation period, oocytes from small, medium, and large follicles were stained with 200 nM MitoTracker Deep Red (Invitrogen, Carlsbad, CA; Cat. M22426) in Medium‐199 containing 1% sodium pyruvate and 0.1% gentamicin for 40 min at 38.5°C. Oocytes were washed three times in 0.1% polyvinylpyrrolidone‐treated PBS (PBS‐PVP) and fixed for 20 min in 4% paraformaldehyde. Following fixation, oocytes were co‐stained with 10 µg/mL Nile red and 1 µg/mL Hoechst 3342 (Thermo Fisher, Cat. 62249) for 15 min for lipid and nuclear labeling, respectively. A Nile red stock solution was prepared using 1 mg Nile red (Sigma‐Aldrich, Cat. N3013) powder diluted in 1 mL DMSO, and the stock was further diluted in PBS‐PVP to a final concentration of 10 µg/mL. Nile red fluorescence was used as a quantitative proxy for neutral lipid droplet abundance and spatial distribution rather than lipid species composition. While this approach does not resolve lipid class or saturation state, it remains a widely applied method for comparative assessment of lipid accumulation in bovine oocytes and embryos. Oocytes were mounted on slides over 10 μL of SlowFade Gold antifade reagent (Life Technologies), covered with a coverslip, and kept at 4°C until imaging. Digital images for individual oocytes were acquired using a Keyence BZ‐X800 microscope (Keyence, Itasca, IL) using mCherry and Cy7 filters at 40X magnification. Digital images were analyzed using Fiji software version 2.9.0 (NIH, Bethesda, MD). Mitochondrial and lipid distribution were evaluated in 297 oocytes derived from small, medium, and large follicles over four independent replicates (Figures 1 and 2), a visual representation of follicle sizes is depicted in Figure 1A. Active mitochondria and lipid content were evaluated in 305 oocytes derived from small, medium, and large follicles over four independent replicates (Figure 3A,B).

Characterization of organelle distribution was performed as reported elsewhere (Machatkova et al. 2012; Stojkovic et al. 2001; Wang et al. 1997; Yoshida et al. 1993). Organelle distribution patterns were determined by confocal imaging and classified as diffuse, semi‐peripheral, peripheral, or other. Diffuse distribution was characterized by organelles being evenly dispersed throughout the cytoplasm. Semi‐peripheral distribution accounted for organelle aggregation without clustering in the central cytoplasm. Peripheral distribution was characterized by organelles primarily located near the oocyte's plasma membrane. Representative images of organelle distribution are presented in Figures 1A and 2A.

For cortical granule and transzonal projection staining, COCs were fixed at the respective maturation period from small, medium, and large follicles for 30 min in 4% paraformaldehyde. Cortical granule staining was performed based on protocols previously reported (Chasombat et al. 2015; Izadyar et al. 1998) with a few modifications. Briefly, following fixation, oocytes were permeabilized in a 0.01% Triton X‐100 (Sigma‐Aldrich, Cat. T8787) in PBS for 1 h at room temperature, washed thrice in PBS‐PVP, and blocked in 0.1% BSA, 0.75% glycine (Honeywell Fluka, Morris Planes, NJ, USA), and 0.2% sodium azide (Fisher Scientific) for 30 min at room temp. Thereafter, COCs were washed thrice in PBS‐PVP and stained in 10 µg/mL FITC‐conjugated lectin (peanut agglutinin; PNA; Vector Laboratories, Newark, CA, USA; Cat. FL‐1071) for 45 min at room temperature in the dark. COCs were again washed thrice in PBS‐PVP and co‐stained in 0.165 µL/mL Alexa Fluor 568 Phalloidin (Invitrogen, Cat. A12380) to visualize TZPs and Hoechst 33342 for 30 min at room temperature in the dark. Finally, COCs were washed and mounted on slides over 10 μL of SlowFade Gold antifade reagent (Invitrogen, Cat. S36938), covered with a coverslip, and kept at 4°C until imaging. Images for individual COCs were acquired using a Nikon C2+ confocal laser scanning microscope (Meville, NY). Fiji software was used to count individual transzonal projection filaments present at three cross‐sections for each oocyte. Only those with an obvious connection between the cumulus cell and oocyte were considered, excluding those with clear breaks between the two. Quantifications of TZPs were performed from three optical sections per oocyte obtained at the equatorial plane and ±5 µm above and below the equator. TZPs were counted only when a continuous actin filament connecting cumulus cells to the oocyte across the zona pellucida was visible. Because the cross‐sectional perimeter varies with oocyte diameter, TZP counts are presented as absolute values and should be interpreted cautiously. Cortical granules were evaluated at an equatorial plane to determine distribution, with three slices evaluated per oocyte, with a total of 210 oocytes across all sizes over four independent replicates.

### 2.5 Cumulus Cell Quantitative RT‐PCR

Total RNA was isolated from cumulus granulosa cell pools from oocytes derived from small, medium, and large follicles matured for 0, 12, or 24 h (*n* = 3 size/timepoint) using TRIzol reagent (Thermo Fisher Scientific) according to the manufacturer's protocol. Total RNA was treated with DNase I (Qiagen, Valencia, CA) during purification to remove genomic contamination. First‐strand cDNA was synthesized from total RNA using LunaScript RT SuperMix (New England Biolabs, Ipswich, MA) using the following conditions: primer annealing at 25°C for 2 min; cDNA synthesis at 55°C for 10 min; and heat inactivation at 95°C for 1 min.

Real‐time PCR was used to evaluate the expression of genes related to steroidogenesis, oocyte competence, glycolysis, and mTOR pathway in cumulus cells from small, medium, and large follicles at 0, 12, and 24 h of maturation. Real‐time PCR was performed using SYBR Green qPCR Master Mix (GlpBio, Montclair, CA) and a CFX96 Touch Real‐Time PCR System (Bio‐Rad Laboratories). Primer sequences for genes aromatase (*CYP19A*), steroidogenic acute regulatory protein (*STAR*), pappalysin‐1 (*PAPPA*), insulin‐like growth factor binding protein 4 (*IGFBP4*), epidermal growth factor receptor (*EGFR*), fibroblast growth factor 2 (*FGF2*), glucose transporter 1 (*SLC2A1*), glucose transporter 3 (*SLC2A3*), lactate dehydrogenase (*LDHA*), hexokinase 2 (*HK2*), and eukaryotic translation initiation factor 4E‐binding protein 1 (*4EBP1*) are listed in Table 1. Primers were validated using a 7‐log dilution curve, and peptidylprolyl isomerase A (*PPIA*) and beta‐actin (*ACTB*) were used as reference genes. All samples were analyzed in duplicate in 10 µL reactions consisting of 0.5 µL of 10 µM primers, 5 µL of supermix, 1 µL nuclease‐free water, and 3 µL of cDNA (5 ng) using the following PCR conditions: activation at 95°C for 2 min; 40 cycles of 95°C for 5 s; 60°C for 30 s; and 72°C for 30 s. Gene expression was calculated relative to reference genes for each sample as fold change using 2^−ΔCT^.

**Table 1:** Primer sequences used in quantitative real‐time PCR.

Gene	Accession No.	Primer Sequences (5′‐ 3′)	
		Forward	Reverse
*PPIA* ^a^	NM_178320.2	AAGGTGAAAGAGGGCATGAATA	CATTTGTCCACAGTCAGCAATG
*ACTB* ^b^	NM_173979.3	GGCGCTTGACTCAGGATTTA	CGGCCACACTGTAGAACTTT
*CYP19A1* ^c^	NM_174305.1	CTCATGGATTTGCAGCCTACTA	TGCTGGTGGCTTGTCTTT
*STAR* ^d^	NM_174189.3	GGACGAGGTGCTGAGTAAAG	CGCTCCACAAGCTCTTCATA
*PAPPA* ^e^	XM_024996354.2	CCTCACCCTGCTCTGATTTAC	GCAATCTCCACCGTCATAGTT
*IGFBP4* ^f^	NM_174557.4	GGGTGTGTCTGTGTGTATGT	GGCTCTCCTGGGCTATCTA
*EGFR* ^g^	XM_002696890.6	GGAGAAGGAGTATCATGCAGAAG	CTCCCAAACAGTGACTCCATAG
*FGF2* ^h^	NM_174056.4	CTGTGTCTAGCCTGCTTGTATG	CTATCCAGGCAGTGCTGATTT
*SLC2A1* ^i^	NM_174602.2	TGCTGAGCGTCATCTTCATC	TCTCCTCGTTGCGGTTAATG
*SLC2A3* ^j^	NM_174603.3	CAGGAGATGAAGGATGAGAGTATG	TAATGATGGGTTGCCGGTAG
*LDHA* ^k^	NM_174099.2	GCCTGTGCCATCAGTATCTT	CCATCATCTCTCCCTTCAGTTTAT
*HK2* ^l^	XM_015473383.2	TGAGATGGAGAGCCAGATCTAT	CCCAAAGGGAGCTTCTTATCTT
*4EBP1* ^m^	NM_001077893.2	TTTGAGATGGACATTTAAAGGGC	CTTGCATAAGGCCTGGCTG

^a^ *PPIA*: peptidylprolyl isomerase A;

^b^ *ACTB*: beta‐actin;

^c^ *CYP19A1*: aromatase;

^d^ *STAR*: steroidogenic acute regulatory protein;

^e^ *PAPPA*: pappalysin‐1;

^f^ *IGFBP4*: insulin like growth factor binding protein 4;

^g^ *EGFR*: epidermal growth factor receptor;

^h^ *FGF2*: fibroblast growth factor 2;

^i^ *SLC2A1*: glucose transporter 1;

^j^ *SLC2A3*: glucose transporter 3;

^k^ *LDHA*: lactate dehydrogenase A;

^l^ *HK2*: hexokinase 2;

^m^ *4EBP1*: eukaryotic translation initiation factor 4E‐binding protein 1.

### 2.6 IVP

IVP was performed to evaluate the developmental potential of oocytes derived from small, medium, and large follicles. Maturation medium composition is detailed in the first experiment. Fertilization medium was Tyrode's albumin lactate pyruvate fertilization medium (IVF‐TALP), and culture medium was synthetic oviduct fluid culture medium (SOF‐BE2). Media composition and IVP followed previously described procedures (Stoecklein et al. 2021; Tríbulo et al. 2019). For fertilization, sperm were purified from frozen‐thawed straws using a gradient (Isolate, FUJIFILM Irvine Scientific) and diluted to a final concentration of 1 × 10^6^ in a 35 mm Petri dish containing 1.7 mL IVF‐TALP and 80 µL of penicillamine‐hypotaurine‐epinephrine (PHE) solution per dish containing expanded COCs. Fertilization occurred over a 16 h period at 38.5°C, 5% CO_2_, and humidified air.

Presumptive zygotes were denuded by vortexing groups for 5 min in 500 µL of HEPES‐TALP containing 10,000 U/mL hyaluronidase. Groups of approximately 25 zygotes were cultured in 500 µL of pre‐equilibrated culture medium overlaid with mineral oil for 8 d at 38.5°C, 5% O_2_, 5% CO_2_, and the balance N_2_. Cleavage was assessed at day 3 post‐insemination (day 0 = day of insemination) by visual inspection under a stereomicroscope. Presumptive zygotes that had undergone at least one cell division and contained two or more visible blastomeres were classified as cleaved. Cleavage rate was calculated as the proportion of cleaved embryos relative to the total number of presumptive zygotes placed in culture. Similarly, blastocyst rates or the percent of zygotes in culture that develop into blastocysts were determined at day 7.5 post‐insemination. A total of 638 presumptive zygotes were produced in seven independent replicates.

### 2.7 Cryopreservation of Embryos by Slow Freezing

On day 7.5 post‐insemination, blastocysts from small, medium, and large follicles were grouped according to their stage and quality grade using IETS standards (Barfield et al. n.d.). Grade 1, stage 7 blastocysts were washed three times in HEPES‐TALP to remove residual culture medium. Blastocysts were then transferred to a drop of freezing medium (HEPES‐TALP, 1.5 M ethylene glycol, and 0.1 M sucrose) and allowed to equilibrate by sinking to the bottom of the drop. Embryos were packaged in 0.25 cc straws by adding a column of freezing medium, followed by approximately 5 mm of air, another column of freezing medium containing two embryos of the same stage and grade, about 5 mm of air, and a final column of freezing medium. Loaded straws were then placed in the freezing machine (EFT‐3002 Portable Embryo Freezer) pre‐cooled to −6.5°C. Individual straws were seeded by placing a copper rod in liquid nitrogen and positioning it on each side of the top column of freezing medium until ice crystals formed. Straws were subjected to a temperature gradient of −0.5°C/min until reaching −30°C. Straws were held at −30°C for 10 min, immersed in liquid nitrogen, and stored until analysis.

To thaw embryos, straws were held at room temperature for 3 s and then placed in a 35°C water bath for 30 s. Embryos were washed three times in HEPES‐TALP with no holding time between washes, and transferred to SOF‐BE2 supplemented with 10% FBS. Re‐expansion was evaluated at 24 h post‐thaw by visual assessment under a stereomicroscope; blastocysts displaying a visible, refilled blastocoel cavity were classified as re‐expanded. A total of 72 embryos were evaluated for re‐expansion 24 h post‐thaw across four independent replicates.

### 2.8 Lipid Droplet Evaluation in Bovine Blastocysts

Blastocysts were produced as described above. On d 7.5, blastocysts were fixed for 20 min in 4% paraformaldehyde. Following fixation, blastocysts were co‐stained with 10 µg/mL Nile red and 1 µg/mL Hoechst 3342 for 15 min for lipid and nuclear labeling, respectively. Oocytes were mounted on slides over 10 μL of SlowFade Gold antifade reagent, covered with a coverslip, and evaluated with a Keyence BZ‐X800 microscope using mCherry filter and 40X magnification. Lipid content was assessed in 70 blastocysts produced over four independent replicates. Lipid droplet content was quantified from Nile red fluorescence images using Fiji (ImageJ, NIH). For each blastocyst, a region of interest (ROI) was manually drawn around the oocyte boundary at the equatorial plane. Mean fluorescence intensity within the ROI was measured in the mCherry channel, which captures Nile red emission corresponding to neutral lipid droplets. Background fluorescence was subtracted using a cell‐free region of the same image. Lipid content is reported as mean Nile red fluorescence intensity (arbitrary units). This approach provides a relative, quantitative comparison of neutral lipid abundance across groups under identical staining and imaging conditions.

### 2.9 Statistical Analyses

All statistical analyses were performed using SAS (Version 9.4; SAS Institute Inc., Cary, NC). Oocyte mitochondrial membrane potential, lipid content, transzonal projection count, and organelle distribution were analyzed by ANOVA using the GLIMMIX procedure in SAS. The model included follicle size, time, and the interaction of size and time. Least‐squares means comparisons between follicle sizes and maturation time were performed using the PDIFF option with Tukey's adjustment for multiple comparisons. Embryo development and cryosurvival were evaluated by ANOVA using the GLM procedure of SAS. Gene expression was analyzed using a generalized linear model including follicle size, time, and the interaction of size and time as fixed factors in the model. Residual diagnostics were examined for all ANOVA models, confirming that assumptions of normality and homogeneity of variance were adequately met. Statistical significance was denoted by *p*‐values ≤ 0.05, with results presented as least‐squares means ± standard error of the mean.

## 3 Results

### 3.1 Mitochondria Distribution and Activity in Oocytes From Small, Medium, and Large Follicles

At 0 h of maturation, oocytes from small follicles (27.78 ± 7.47%) exhibited a higher percentage of peripheral mitochondrial distribution compared to those from medium (3.13 ± 3.01%; *p* = 0.03) and large follicles (3.33 ± 3.28%; *p* = 0.03; Figure 1C). At 12 h, oocytes from large follicles (86.97 ± 7.02%) showed a greater percentage of diffuse mitochondrial distribution than those from medium follicles (57.14 ± 8.37%; *p* = 0.03), but not compared to those from small follicles (74.47 ± 6.36%; *p* = 0.25; Figure 1D). By 24 h, no significant differences in mitochondrial distribution were observed among follicle sizes (*p* > 0.05; Figure 1E).

Active mitochondria content was also evaluated by follicle size (*p* = 0.54), time (*p* < 0.0001), and their interaction (*p* = 0.11). Quantity of active mitochondria did not differ among follicle sizes at 0 h (*p* > 0.05; Figure 1F). At 12 h, oocytes from medium follicles (670.54 ± 87.03) had fewer active mitochondria than those from small follicles (921.41 ± 78.05; *p* = 0.02) but not compared to large follicles (712 ± 110.38; *p* = 0.59). No significant differences were detected at 24 h (*p* > 0.05; Figure 1F).

### 3.2 Lipid Distribution and Content in Oocytes from Small, Medium, and Large Follicles

Lipid distribution followed similar patterns to mitochondrial distribution. At 0 h, oocytes from small follicles (27.78 ± 7.47%) displayed more peripheral lipid localization compared to those from medium (3.13 ± 3.01%; *p* = 0.03) and large follicles (3.33 ± 3.28%; *p* = 0.03; Figure 2B). At 12 h, oocytes from large follicles (86.97 ± 7.02%) exhibited more diffuse lipid distribution than those from medium follicles (57.14 ± 8.37%; *p* = 0.03), but not small follicles (74.47 ± 6.363%; *p* = 0.25; Figure 2C). No differences in lipid distribution were observed by 24 h (*p* > 0.05; Figure 2D).

Lipid droplet content was also evaluated by follicle size (*p* = 0.21), time (*p* = 0.15), and their interaction (*p* = 0.001). At 0 h, oocytes from large follicles (784.67 ± 114.87) had lower lipid content than those from medium follicles (1102.94 ± 109.52; *p* = 0.02), but not small follicles (839.59 ± 103.43; *p* = 0.73; Figure 2E). At 12 h, lipid content was higher in oocytes from large follicles (1136.26 ± 131.19) compared to those from small follicles (688.17 ± 92.76; *p* = 0.007), but not medium follicles (877.57 ± 103.43; *p* = 0.07). At 24 h, oocytes from large follicles (795.50 ± 128.43) had the lowest lipid content compared to medium (1174.42 ± 113.00; *p* = 0.04) and small follicles (1205.39 ± 104.86; *p* = 0.008), while oocytes from small follicles exhibited the highest lipid content (Figure 2E).

### 3.3 Cortical Granule Distribution

No differences in cortical granule distribution were observed among oocytes from different follicle sizes at 0 h (*p* > 0.05; Figure 3B). While cortical granules generally migrated peripherally during maturation, no significant differences among groups were detected at 12 or 24 h (*p* > 0.05; Figure 3C,D).

### 3.4 Transzonal Projection Counts

**Figure 4:**
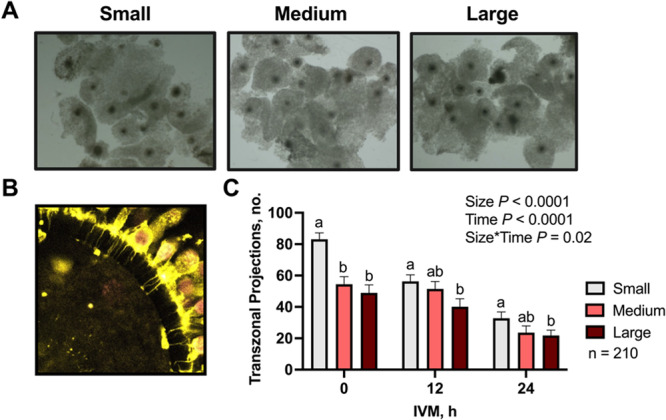
Transzonal projections in COCs from small, medium, and large follicles during IVM. (A) Representative images of COCs from all three follicle sizes following 24 h IVM. (B) Visualization of TZPs connecting cumulus cells to the oocyte crossing the zona pellucida. (C) Quantification of intact TZPs at 0, 12, and 24 h IVM (*n* = 210 oocytes; ~23 oocytes per follicle size/timepoint). Oocytes from large and medium follicles had fewer intact TZPs than those from small follicles (*p* < 0.0001). At 12 h, oocytes from large follicles had fewer TZPs than those from small (*p* = 0.01), with no difference compared to medium (*p* = 0.1). At 24 h, oocytes from large follicles continued to show fewer TZPs than those from small follicles (*p* = 0.04).

A significant effect for size (*p* < 0.0001), time (*p* < 0.0001), and their interaction (*p* = 0.02) was found. At 0 h, oocytes from small follicles exhibited a higher number of TZPs (83.1 ± 4.1) compared to those from medium (54.50 ± 4.80; *p* < 0.0001) and large follicles (49.00 ± 5.10; *p* < 0.0001; Figure 4C). At 12 h, TZP numbers remained higher in oocytes from small follicles (56.30 ± 4.20) than in those from large follicles (40.10 ± 5.10; *p* = 0.01), but not compared to medium follicles (51.50 ± 4.80; *p* = 0.10). This trend persisted at 24 h, with lower TZP numbers in oocytes from large follicles (21.80 ± 3.50) compared to small follicles (32.80 ± 4.00; *p* = 0.04), while medium and large follicle groups did not differ (23.60 ± 4.30; *p* = 0.73).

### 3.5 Embryo Development, Cryosurvival, and Lipid Content

**Figure 5:**
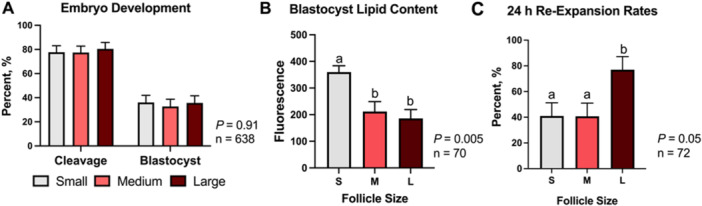
Embryo development, lipid content, and cryosurvival. (A) Cleavage and blastocyst rates for embryos derived from small, medium, and large follicles (*n* = 638 presumptive zygotes). No differences were observed in cleavage or blastocyst rates among follicle sizes (*p* = 0.91). (B) Lipid droplet content in day 7.5 blastocysts (*n* = 70 blastocysts; ~23 blastocysts per follicle size). Blastocysts derived from small follicle oocytes had greater lipid content than those from medium (*p* = 0.001) or large (*p* < 0.0001) follicles. (C) Re‐expansion rates of blastocysts 24 h after thawing following slow‐freezing (*n* = 72 blastocysts; ~24 blastocysts per follicle size). Blastocysts from large follicles had greater post‐thaw re‐expansion compared to small (*p* = 0.05) and medium (*p* = 0.05) follicles.

Cleavage (*p* = 0.91) and blastocyst (*p* = 0.91) rates did not differ among embryos derived from small, medium, or large follicles (Figure 5A). However, blastocysts from small follicles had higher lipid content (360.1 ± 33.2) than those from medium (211.7 ± 37.1; *p* = 0.001) and large follicles (186.0 ± 33.2; *p* < 0.0001; Figure 5B). Re‐expansion rates 24 h post‐thaw were significantly greater in blastocysts derived from large follicle oocytes (77.0 ± 10.2%) compared to those from small (41.0 ± 10.2%; *p* = 0.05) and medium (41.0 ± 10.2%; *p* = 0.05) follicles (Figure 5C).

### 3.6 Cumulus Cell Gene Expression

**Figure 6:**
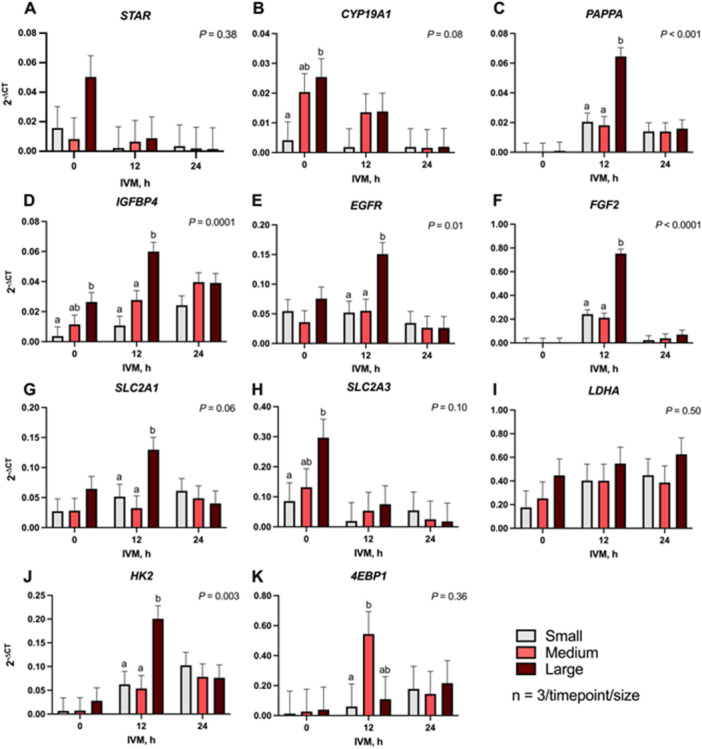
Gene expression in cumulus cells associated with oocytes from small, medium, and large follicles. Expression of steroidogenic, growth factor, metabolic, and signaling genes evaluated at 0, 12, and 24 h IVM (*n* = 3 pool replicates/size/timepoint). (A) *STAR*: No differences observed across follicle sizes at any time point (*p* > 0.05). (B) *CYP19A1*: Higher in cumulus cells from large follicles compared to small follicles at 0 h (*p* = 0.02); no differences at 12 or 24 h. (C) *PAPPA*: No differences at 0 or 24 h; expression was greater in cumulus cells from large follicles compared to small and medium follicles at 12 h (*p* < 0.0001). (D) *IGFBP4*: Higher expression in large follicles than medium follicles at 0 h (*p* = 0.02) and greater than small (*p* = 0.002) and medium (*p* < 0.0001) follicles at 12 h; no differences at 24 h IVM. (E) *EGFR*: No differences at 0 h; higher expression in large follicles compared to small (*p* = 0.0021) and medium (*p* = 0.0006) follicles at 12 h; no differences at 24 h IVM. (F) *FGF2*: No differences at 0 or 24 h IVM; higher expression in large follicles than small and medium follicles at 12 h IVM (*p* < 0.0001). (G) *SLC2A1*: No differences at 0 or 24 h; greater expression in large follicles at 12 h compared to small (*p* = 0.01) and medium (*p* = 0.003) follicles. (H) *SLC2A3*: Higher expression in large follicles compared to small follicles at 0 h (*p* = 0.03); no differences at 12 or 24 h. (I) *LDHA*: No differences at any time point (*p* > 0.05). (J) *HK2*: No differences at 0 or 24 h; greater expression in large follicles at 12 h compared to small (*p* = 0.002) and medium (*p* = 0.001) follicles. (K) *4EBP1*: No differences at 0 or 24 h; higher expression in medium follicles compared to small follicles at 12 h (*p* = 0.04).

Expression of *STAR* did not differ among follicle sizes at any time point (*p* > 0.05; Figure 6A). *CYP19A1* expression was 6.11‐fold lower in cumulus cells from small follicles compared with large follicles at 0 h (*p* = 0.02), with no differences detected at 12 or 24 h (Figure 6B).

*PAPPA* expression did not differ among follicle sizes at 0 or 24 h (*p* > 0.05), but was 3.15‐fold higher in cumulus cells from large follicles compared with small and medium follicles at 12 h (*p* < 0.0001; Figure 6C). Expression of *IGFBP4* was consistently higher in cumulus cells from large follicles. At 0 h, expression was 3.12‐fold higher compared with medium follicles (*p* = 0.02) and 2.16‐fold higher compared with small follicles (*p* = 0.002). At 12 h, *IGFBP4* expression was 2.16‐fold higher in large compared with small follicles (*p* = 0.002) and 5.58‐fold higher than medium follicles (*p *< 0.0001). No differences were detected at 24 h (Figure 6D). *EGFR* expression did not differ at 0 h but was 2.9‐fold higher in cumulus cells from large follicles compared with small (*p* = 0.002) and medium follicles (*p* = 0.0006) at 12 h. No differences were observed at 24 h (Figure 6E). Similarly, *FGF2*, *SLC2A1*, and *HK2* expression did not differ among follicle sizes at 0 or 24 h, but were significantly higher in cumulus cells from large follicles at 12 h compared with both small and medium follicles (*p* < 0.05; Figure 6F,G,J). Expression of *SLC2A3* was 3.5‐fold higher in cumulus cells from large follicles compared with small follicles at 0 h (*p* = 0.03), with no differences detected at later time points (Figure 6H). *LDHA* expression did not differ among follicle sizes at any time (*p* > 0.05; Figure 6I). Expression of *4EBP1* did not differ at 0 or 24 h but was eightfold higher in cumulus cells from medium follicles compared with small follicles at 12 h (*p* = 0.04; Figure 6K).

## 4 Discussion

This study demonstrates that follicle size significantly influences oocyte cytoplasmic maturation, lipid metabolism, and embryo cryosurvival in‐vitro. While IVM promoted progression toward maturation across all follicle sizes, early differences in mitochondrial organization, lipid accumulation, TZP dynamics, and cumulus cell gene expression indicate that follicular origin establishes distinct metabolic states that are not fully erased by standardized culture conditions. These early differences likely contribute to downstream variation in embryo quality, particularly with respect to cryotolerance.

Mitochondrial redistribution from peripheral to diffuse localization is a hallmark of cytoplasmic maturation and oocyte competence (Ferreira et al. 2009; Reader et al. 2017). In this study, mitochondrial distribution was relatively similar across follicle size by the end of maturation. Additionally, mitochondrial changes during oocyte maturation vary across species. In rodents, oocytes in the germinal vesicle stage have mitochondrial aggregation in the perinuclear region and become evenly distributed by the end of MII (Kirillova et al. 2021). Lipid distribution followed a similar trajectory to mitochondria, as these two work together, creating metabolic units to provide the oocyte with energy. Interestingly, oocytes from large follicles exhibited lower lipid droplet accumulation by the end of IVM, while oocytes and embryos from small follicles retained significantly more lipids, consistent with greater fatty acid exchange between cumulus cells and the oocyte in smaller follicles. Despite similar spatial patterns of mitochondrial and lipid redistribution, functional outcomes differed among follicle sizes, indicating that organelle localization alone does not necessarily reflect equivalent metabolic activity or substrate utilization under in‐vitro conditions. Differences in lipid content between in‐vitro and in‐vivo matured oocytes have been linked to the static nature of IVM media compared to the dynamic follicular environment (Moorey et al. 2021). The elevated lipid content observed in oocytes and blastocysts from small follicles aligns with this paradigm and may contribute to reduced cryotolerance due to excess intracellular lipid storage.

While the current results indicate that IVM conditions can partially homogenize developmental competence across follicle sizes, they also reinforce long‐standing evidence that oocytes from larger follicles possess superior intrinsic quality, particularly in post‐thaw survival and lipid remodeling (Hendriksen et al. 2000; Lonergan et al. 1994; Sarwar et al. 2020). Even under identical IVM conditions, embryos derived from large follicles exhibited enhanced cryosurvival, suggesting that follicle‐associated metabolic advantages persist beyond maturation. Because ovaries were obtained from an abattoir without endocrine profiling, follicles could not be classified by wave stage or functional status. Thus, some large follicles may have been preovulatory or regressing. However, this heterogeneity reflects the biological variability inherent to slaughterhouse IVP systems. Importantly, despite this variability, follicle‐size‐dependent differences in lipid remodeling and cryosurvival emerged consistently across replicates.

The communication between cumulus cells and the oocyte is mediated by TZPs, actin‐rich projections that cross the zona pellucida, establishing direct contact between them and regulating maturation through bidirectional molecule exchange. While TZP numbers increase during folliculogenesis, TZPs regress as maturation progresses and cumulus cell expansion occurs. This leads to a reduction in fatty acid transfer, and meiotic arrest signaling is terminated (Clarke 2022; Del Collado et al. 2017; Macaulay et al. 2014). Oocytes from large follicles had fewer TZPs and lower lipid content by the end of maturation, possibly reflecting earlier and more complete TZP retraction along with reduced lipid uptake. Because TZPs were quantified as absolute counts rather than normalized to zona perimeter, differences in oocyte diameter across follicle sizes may contribute to observed differences. Future studies should evaluate TZP density to distinguish structural scaling effects from true differences in intercellular connectivity. This study focused on descriptive and functional endpoints of cytoplasmic maturation. While differences in TZP number and lipid dynamics were observed, direct regulators of actin remodeling, fatty acid transport, and β‐oxidation were not evaluated. Additionally, TZPs were quantified as absolute counts rather than normalized densities, which may partially reflect differences in oocyte size.

Gene expression in cumulus cells revealed transient differences that largely normalized by 24 h. At 0 h, *CYP19A1* (aromatase) was lower in small‐follicle cumulus cells, potentially reflecting less active steroidogenesis. Similarly, *PAPPA* and *IGFBP4*, key regulators of IGF bioavailability, were significantly elevated in large‐follicle cumulus cells at 12 h but equalized by 24 h. These findings suggest that large follicles provide a more robust early endocrine and growth factor environment. Meanwhile, genes associated with EGF signaling (e.g., *EGFR*, *FGF2*) also peaked midway through maturation in large‐follicle COCs, in line with previous reports that EGF‐like growth factors are more abundant in preovulatory follicles (Richani and Gilchrist 2018). While expression of *EGFR* and *FGF2* was greater in large‐follicle cumulus cells at 12 h, no differences persisted at 24 h, indicating transient follicle‐size‐dependent expression. Glucose metabolism‐related genes, including *SLC2A1*, *SLC2A3*, and *HK2*, followed a similar trend, with higher expression in large‐follicle cumulus cells early in maturation and no significant differences by 24 h. Expression of *STAR*, encoding the steroidogenic acute regulatory protein responsible for mitochondrial cholesterol import and progesterone biosynthesis, did not differ among follicle size groups at any time point. This null result may reflect functional redundancy in steroidogenic regulation under in‐vitro conditions, or alternatively, that *STAR* expression in cumulus cells is primarily regulated by LH signaling, which is absent under standard IVM conditions. Similarly, *LDHA* expression, encoding the enzyme responsible for converting pyruvate to lactate in glycolysis, did not differ among groups, suggesting that baseline glycolytic output in cumulus cells is relatively uniform regardless of follicle origin. In contrast, *4EBP1*, a downstream effector of the mTOR signaling pathway, was transiently elevated in cumulus cells from medium follicles compared to small follicles at 12 h IVM. This midpoint increase in *4EBP1* suggests transient mTOR pathway activity among follicle size classes during peak cumulus expansion. These genes are central to glycolysis and pyruvate production in cumulus cells, which in turn supply energy substrates to the oocyte via TZPs. Though early transcriptional differences may reflect metabolic readiness, the normalization by 24 h underscores the capacity of IVM conditions to partially compensate for initial disparities.

From a broader perspective, these results highlight that follicle size remains an important biological and practical selection criterion. Although cleavage and blastocyst rates were ultimately similar across follicle sizes, embryos from large follicles demonstrated superior cryotolerance. This outcome aligns with recent reviews, which emphasize that follicle size reflects early metabolic programming that IVM cannot fully account for (Aguila et al. 2020; García‐Guerra et al. 2025). These intrinsic differences influence lipid utilization and membrane composition, ultimately determining post‐thaw survival and long‐term embryo performance.

Additionally, while variation in IVP success among laboratories is often attributed to differences in IVM media composition (e.g., serum supplementation or specific growth factors), the present data suggest that oocyte selection based on follicle size has a greater influence on embryo cryosurvival. Thus, refining selection criteria along with media optimization may yield more reproducible studies and higher‐quality embryos. Finally, from a translational standpoint, systematic selection for larger follicles before ovum pick‐up could enhance commercial IVP efficiency by increasing the proportion of embryos with greater cryosurvival potential. This approach could subsequently improve cost‐effectiveness, facilitate genetic dissemination, and increase consistency in embryo transfer outcomes across programs.

Despite these early differences, cleavage and blastocyst rates did not differ among follicle sizes, suggesting that nuclear and cytoplasmic maturation were ultimately sufficient for development. However, blastocysts derived from large‐follicle oocytes exhibited superior cryosurvival, with higher re‐expansion rates after slow freezing, consistent with their lower lipid content. The use of fetal bovine serum during IVM and post‐thaw culture represents an important limitation when interpreting lipid phenotypes. Accordingly, the present findings should be interpreted as relative differences among follicle size groups under identical serum‐supplemented conditions, rather than as absolute measures of intrinsic lipid metabolism. Future studies employing defined IVM/IVC systems and direct assessment of lipid transport pathways will be essential to dissect follicle‐intrinsic effects from culture‐induced lipid flux. While standard IVM conditions promote partial normalization of cytoplasmic and molecular maturation markers, follicle size establishes early metabolic programming that persists through maturation and influences lipid metabolism, preimplantation development, and embryo cryosurvival. These findings underscore the potential value of follicle size as a biologically informed selection criterion in IVP to enhance post‐thaw embryo viability. These findings emphasize the potential value of follicle size as a selection criterion in IVP to enhance post‐thaw viability.

## 5 Conclusions

ARTs such as IVP offer opportunities for genetic advancement and reproductive efficiency in cattle. However, optimizing oocyte selection remains a critical determinant of embryo quality and yield. This study indicates that follicle size influences the trajectory of cytoplasmic maturation, including mitochondrial and lipid remodeling, cumulus cell metabolism, and TZP dynamics, all of which impact embryo cryosurvival. A consistent feature across outcomes was the emergence of follicle size‐dependent differences during early to mid maturation that largely converged by 24 h IVM. This pattern suggests that while standard IVM conditions promote morphological and transcriptional normalization, early metabolic programming associated with follicle size persists and influences downstream outcomes such as lipid remodeling and cryotolerance.

Oocytes from larger follicles exhibit advanced cytoplasmic maturation and reduced lipid accumulation, leading to embryos with enhanced post‐thaw survival. These findings support the inclusion of follicle size as a biologically informed selection criterion in IVP systems and provide a framework for developing targeted strategies to improve oocyte competence, embryo quality, and cryosurvival in both research and commercial setting.

## Author Contributions

**Briza Castro:** conceptualization, investigation, writing – original draft, methodology, validation, visualization, writing – review and editing, formal analysis, data curation. **Paula Rodriguez‐Villamil:** writing – review and editing, visualization, formal analysis, conceptualization, methodology, resources, software. **M. Sofia Ortega:** conceptualization, investigation, funding acquisition, writing – review and editing, visualization, methodology, formal analysis, data curation, supervision, resources.

## Ethics Statement

Ethical review and approval were not required for this study.

## Conflicts of Interest

The authors declare no conflicts of interest.

## Data Availability

All data is included in the main manuscript. Any additional data that support the findings of this study are available from the corresponding author upon reasonable request.
